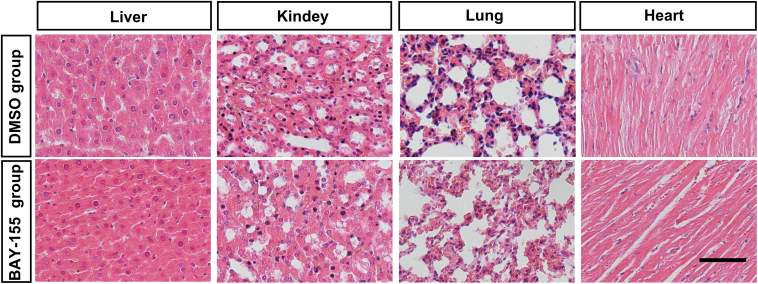# Menin facilitates the cell proliferation of bladder cancer via modulating the TFAP2C/β-catenin axis

**DOI:** 10.1016/j.gendis.2025.101565

**Published:** 2025-02-20

**Authors:** Qing Shi, Xiang Pan, Shiheng Zhang, Mengyuan Wu, Meiqi Xu, Yun-Qi Li, Li Zhong, Zi-Qi Wang, Wanhai Xu, Yakun Luo

**Affiliations:** a NHC Key Laboratory of Molecular Probes and Targeted Diagnosis and Therapy, Harbin Medical University, Harbin, Heilongjiang 150001, China; b Chongqing Key Laboratory of Development and Utilization of Genuine Medicinal Materials in Three Gorges Reservoir Area, Faculty of Basic Medical Sciences, Chongqing Three Gorges Medical College, Wanzhou, Chongqing 404120, China; c National Center for Translational Medicine, Ruijin Hospital, Shanghai Jiaotong University School of Medicine, Shanghai 200233, China; d Centre International de Recherche en Infectiologie (CIRI), Univ Lyon, Inserm, U1111, Université Claude Bernard Lyon 1, CNRS, UMR5308, ENS de Lyon, Lyon 69373, France; e Department of Urology, The Cancer Hospital of Harbin Medical University, Harbin, Heilongjiang 150081, China; f Department of Urology, The Fourth Affiliated Hospital of Harbin Medical University, Harbin, Heilongjiang 150001, China

**Keywords:** β-catenin, Bladder cancer, Cell proliferation, *MEN1*, TFAP2C, Therapeutic target

## Abstract

Bladder cancer (BLCA) is a common malignant tumor of the urinary system, with significant morbidity and mortality rates worldwide. The *MEN1* gene, encoding the menin protein, plays a regulatory role in several cancers. However, the role played by menin in BLCA remains elusive. In this study, our data demonstrated that the expression of menin was significantly up-regulated in BLCA tissues versus normal tissues, and the high expression of menin was strongly correlated with poor prognosis of BLCA patients. *In vitro*, silencing *MEN1* inhibited cell proliferation and induced cell cycle arrest at the G1/S phase in BLCA cells. Furthermore, RNA sequencing analysis revealed that *MEN1* knockdown significantly inhibited the Wnt/β-catenin signaling in BLCA cells. Meanwhile, we further confirmed that β-catenin served as a critical downstream effector of menin in BLCA cells. Mechanically, chromatin immunoprecipitation analysis demonstrated that menin promoted *CTNNB1* (catenin beta 1) transcription through binding to the *CTNNB1* proximal promoter in BLCA cells. Interestingly, menin collaborated with TFAP2C, a regulator of β-catenin in BLCA cells, to enhance the transcription of the *CTNNB1* gene. More intriguingly, BAY-155, a menin molecule inhibitor, inhibited cell growth of BLCA cells both *in vitro* and *in vivo* by suppressing the expression of menin, TFAP2C, and β-catenin. Our current work unveils an important role of the menin in triggering the TFAP2C/β-catenin axis, which contributes to cell proliferation of BLCA cells. Therefore, menin might be served as a new therapeutic target for BLCA.

## Introduction

Bladder cancer (BLCA), being one of the most prevalent malignant tumors in the urinary system, imposes a substantial burden in terms of its frequency and fatality rates.^1^ Despite substantial advancements in clinical diagnostic and therapeutic approaches,^2^ BLCA remains prone to recurrence, metastasis, and the development of drug resistance due to its intricate biological characteristics.^3^ Certain recurrent tumors exhibit limited responsiveness to radiation and chemotherapy, while the effectiveness of biological treatments falls short, resulting in an overall unfavorable prognosis.^4^ The molecular mechanisms underlying tumor metastasis and drug resistance are exceedingly intricate, involving a multitude of factors such as the environment, genetic disorders, or gene mutations.^5^ Currently, there is a lack of ideal biomarkers and effective therapeutic targets in clinical practice. Therefore, it is of paramount importance to delve into the intricate pathogenic mechanisms that underlie the initiation and advancement of BLCA, while concurrently unveiling novel therapeutic targets.

Menin is encoded by the *MEN1* gene, which is mutated in patients with multiple endocrine neoplasia type 1 (MEN1) syndrome.^6^ Functioning as a scaffold protein, menin interacts with a diverse array of transcriptional factors to govern both activating and repressive cellular processes.^7^ Furthermore, it has been documented that menin serves as a facilitator for the presentation of histone H3 lysine 4 (H3K4),^7^^,^^8^ forming a complex alongside mixed lineage leukemia (MLL) family methyltransferases MLL1 and MLL2 responsible for methylation of H3K4.^9^ This intricate assemblage plays a pivotal role in histone methylation and the remodeling of chromatin structure.^10^ Previous studies have unveiled the up-regulation of menin in various malignancies, such as leukemia,^11^ breast,^12^ prostate,^13^ and liver^14^ cancers, implicating its involvement in tumorigenesis and disease progression. However, the role of menin in the progression of BLCA and the underlying mechanism involved remains elusive. More recent studies have shed light on the oncogenic roles of menin's partners, such as MLL^15^ and WDR5,^16^^,^^17^ in promoting the proliferation, recurrence, and cisplatin chemoresistance of BLCA. Therefore, dissecting the specific downstream signaling pathway of menin and these pathway-regulated key genes, which are responsible for mediating menin-induced BLCA growth, will aid in identifying alternative molecular targets for developing novel therapeutic strategies.

More intriguingly, a series of menin small molecule inhibitors (MIs) (such as MI-503, M−525, MI-136, or MI-2) have been reported and used to delve into the multifaceted role of menin across diverse cancer indications in recent years.^13^^,^^18^^,^^19^ Notably, several MIs have exhibited remarkable efficacy in preclinical models^20^ or have recently progressed to phase 1 clinical trials,^21^ particularly in the context of MLL-fused leukemia. Recently, Brzezinka et al reported the distinctive properties of BAY-155 as a selective menin inhibitor.^22^^,^^23^ In addition to its potent inhibitory effects on leukemia cells, BAY-155 has been demonstrated to elicit anti-proliferative effects in various cancer cells, including BLCA cells.^22^ Consequently, further exploration of the functional attributes of BAY-155 and a comprehensive understanding of the underlying mechanisms it harbors in BLCA is imperative for the advancement of targeted therapies against BLCA.

Herein, we unveiled the pivotal role of menin in BLCA and delved into the underlying mechanism in BLCA cells. The results of *in vitro* and *in vivo* experiments provide the evidence for menin to play an oncogenic role in BLCA, indicating that menin is a new diagnostic marker and therapeutic target for BLCA.

## Materials and methods

### Patients and specimens

Tissue samples were obtained from patients who underwent surgery in the Department of Urology, Cancer Hospital of Harbin Medical University, between January 2020 and December 2022, and whose postoperative pathology was confirmed as BLCA. All studies on human specimens were approved by the Ethics Committee of Harbin Medical University (No. 2022-SCILLSC-30).

### Cell culture and reagents

BLCA cell lines, including human immortalized uroepithelial cells (SV-HUC-1) and three BLCA cell lines (T24, 5637, and HT-1197), were purchased from the American Type Culture Collection (ATCC). The cell lines were cultured in RPMI 1640 medium (A1049101, Gibco, USA) supplemented with 10% heat-inactivated fetal bovine serum (16140071, Gibco, USA) and 1% penicillin-streptomycin (C0222, Beyotime, China) at 37 °C and 5% CO_2_. The menin small molecule inhibitor BAY-155 (PC-38218) was purchased from Probechem Biochemicals, Shanghai, China. All cell lines were confirmed to be free from mycoplasma contamination through PCR testing.

### RNA interfering and transfection

The small interfering RNAs (siRNAs) were purchased from Ribo Bio Tech (Guangzhou, China) including siMEN1#1: stB0007071A-1–5; siMEN1#2: stB0007071B-1–5; siTFAP2C#1: siG000007022A-1–5; siTFAP2C#2: siG000007022B-1–5, and the negative-control siRNA (siCtrl, siN0000001). Transient transfection of cells was performed using Lipofectamine 2000 (11668019, Invitrogen, USA) according to the manufacturer's instructions. Protein and RNA extraction were used to verify the knockdown efficiency.

### RNA extraction and quantitative real-time PCR (RT-qPCR) analysis

RNA was extracted using a total RNA extractor (Trizol) kit (B511311, Sangon, China) and reverse transcribed to cDNA using the ReverTra Ace qPCR RT Kit (FSQ-301, Toyobo, Japan). The iTaq Universal SYBR Green (1725124, Bio-Rad) was used to perform RT-qPCR analysis. Relative RNA expression levels were all measured by the ABI 7500 FAST system (Applied Biosystems, USA). Data were normalized to *ACTB* and presented as fold changes. The primers used are listed in Table S1.

### RNA sequencing analysis

Total RNA was extracted by Trizol reagent (B511311, sangon, China). Each RNA sample preparation was carried out with a total of 3 μg RNA as input. RNA was sequenced by NovaSeq sequencer (Illumina, San Diego, CA) using the Illumina Hiseq 4000 platform. A default set of parameters was used for mapping the reads to the reference genome by HISAT2 (version 2.0). RSeQXC (version 2.6.1) was used for alignment statistics. Gene expression analysis was performed using the DESeq2 (version 1.12.4). An enrichment pathway analysis of genes was compiled by DAVID Bioinformatics Resources using Kyoto Encyclopedia of Genes and Genomes (KEGG) pathway databases. The raw data of RNA sequencing were deposited in Sequence Read Archive (SRA) under accession number PRJNA1057809.

### Cell proliferation assay

CCK-8 analysis (Cell Counting Kit-8, HY-K0301, MCE) was performed to evaluate the cell proliferation as the experiment indicated. Briefly, the required cells were plated in a 96-well plate with 3000 cells per well, the day before the experiment. After the cell adhered, CCK-8 was added to the wells, and the absorbance was measured after incubation at 37 °C for 2 h.

### Cell colony formation assay

Cells were seeded in 6-well culture plates at 5 × 10^2^ cells for BLCA cells. Cells were transfected with siRNAs or treated with BAY-155, and cultured for 2 weeks. The ensuing colonies were stained with 0.5% crystal violet. The images of the plates were analyzed using ImageJ software. Each experiment was conducted in triplicate and statistical analyses were performed using the Prism software.

### Protein extraction and western blotting

Total protein extracts from cells were prepared and analyzed as previously described.^24^ The cellular nuclear fractions were separated using NE-PER Nuclear and Cytoplasmic Extraction Reagents (78833, Thermo Scientific, Waltham, Massachusetts, USA). Antibodies used are listed in Table S2.

### Flow cytometry assay

Cells were digested and fixed in ethanol at −20 °C for 2 h as indicated. Then, the cells were incubated with propidium iodide staining reagent (ST512, Beyotime, China) in the dark for 30 min. All samples were evaluated for cell cycle distribution using a flow cytometry instrument (BD Biosciences; San Jose, CA, USA).

### Chromatin immunoprecipitation assay

Chromatin immunoprecipitation (ChIP) assay was performed according to the manufacturer's protocol as previously described.^25^ Briefly, a total of 2 × 10^7^ cells were prepared using the ChIP assay kit (17–295, Millipore, Massachusetts, USA) in accordance with the manufacturer's instructions. Briefly, the cross-linked chromatin was sonicated to yield fragments ranging from 200 to 500 base pairs. Subsequently, the chromatin was immunoprecipitated using antibodies against menin (A300-105A, Bethyl), TFAP2C (ab218107, Abcam), MLL1 (ab234435, Abcam), ASH2L (ab314128, Abcam), β-catenin (ab224803, Abcam), or H3K4me3 (ab8580, Abcam). The DNA fragments were purified using the QIAquick PCR purification kit (28104, Qiagen, Hilden, Germany) and utilized for RT-qPCR reactions with iTaq Universal SYBR Green (1725124, Bio-Rad). Each ChIP assay was independently repeated at least three times. The primers employed for ChIP-qPCR can be found in Table S1.

### Immunohistochemistry assay

Formalin-fixed paraffin-embedded BLCA specimens or xenografts were meticulously prepared and processed for immunostaining, following the established protocol as previously described.^12^ In brief, the blocks were delicately sectioned at a thickness of 4 μm. The tissue on the slides underwent a meticulous dewaxing and hydration process, followed by blockade with 1% bovine serum albumin to ensure optimal conditions. Subsequently, the menin antibody (1:500) was incubated at 4 °C overnight, while the goat-anti-rabbit antibody (1:1000) was incubated at 37 °C for 1 h. The utilization of diaminobenzidine (SignalStain® DAB Substrate Kit, #8059, Cell Signaling Tech) yielded satisfactory staining outcomes. Finally, methyl green was employed for staining, and a series of meticulously selected reagents, including gradient alcohol and xylene, were used for dehydration. A neutral adhesive was applied for sealing, and the tissue was observed under a microscope (Primovert HDcam, ZEISS, Germany).

### Hematoxylin-eosin staining assay

Hematoxylin-eosin staining assays were performed using a hematoxylin-eosin staining kit (G1120, Solarbio, China), according to the supplier's recommendations. Briefly, after the samples underwent deparaffinization and hydration, they were subjected to a 2-min hematoxylin staining followed by a phosphate buffer saline rinse. Subsequently, a 1-min eosin staining was performed, followed by another phosphate buffer saline wash. Finally, the samples were dehydrated, sealed with neutral gum, and observed under a microscope to obtain the images.

### Xenograft tumor model

The Harbin Medical University Animal Care and Use Committee released the approval (No. SYXK2022-014) for the animal experiment. Athymic nude BALB/c male mice (Beijing Vital River Laboratory Animal Technology Co., China) aged 6 weeks received the housing process within a specific pathogen-free environment based on free water and food as well as 12 h light/12 h dark cycle, and they were feed for 7 days to adapt to the environment. One week post acclimatization, the mice received the random separation in four groups (6 mice/group), and the T24 cells (1 × 10^7^ cells/mice) were re-suspended in a 200 μL mixture of 50% phosphate buffer saline/50% Matrigel (BD Biosciences) which was injected subcutaneously into right lower limb back of the mice. Fifteen days later, animals bearing xenograft tumors reaching 45–50 mm^3^ in size were selected and received the treatment or therapies as experimentally indicated. Tumor size was measured using vernier calipers every 7 days after the first injection of siMEN1, siCtrl, BAY-155, or DMSO as indicated. Tumor volumes were calculated using the following formula: (width)^2^ × height/2. After being fed for 42 days, the mice were euthanized, and the tumors were isolated, weighed, photographed, and immediately fixed by adopting 4% paraformaldehyde to conduct the following investigation.

### Data mining analysis

Gene expression data (Transcripts per million, TPM) and the relevant BLCA prognostic and clinical data were downloaded from The Cancer Genome Atlas (TCGA) (https://portal.gdc.cancer.gov/) databases. GSE31684 was downloaded from the Gene Expression Omnibus (GEO) (https://www.ncbi.nlm.nih.gov/geo/).

### Statistical analyses

Results were presented as mean ± standard deviation of three independent tests. Statistical analyses were performed using the GraphPad Prism 6 software (GraphPad Software Inc., San Diego, CA, USA). The differences between the two groups were assessed by student's *t*-test, and comparisons among three or more groups were first assessed by one-way analysis of variance (ANOVA). The cut-off values for survival analysis were determined using the Maxstat (R package). Results with a *p* value of 0.05 or less were considered statistically significant.

## Results

### The oncogenic role played by menin in BLCA

To determine the role of *MEN1* in BLCA, we first analyzed the *MEN1* gene mRNA level in human BLCA tissues, based on the TCGA datasets. As shown in Figure 1A, the up-regulation of *MEN1* was observed in BLCA tissues (*n* = 408) compared with the normal tissues (*n* = 19). Meanwhile, we also found that the high level of *MEN1* in BLCA was positively correlated with the tumorigenesis of stages 1, 2, 3, and 4 (Fig. 1B; Fig. S1). Importantly, based on the data from the GEO datasets (GSE31684 and GSE13507), we found that the overall survival rate of patients with high expression of *MEN1* was significantly lower than that of patients with low expression of *MEN1* (Fig. 1C). To further study the clinical significance of menin expression in BLCA, we analyzed the expression of menin protein in different stages of BLCA by immunohistochemistry assay in total of 90 BLCA patients. The immunohistochemistry results showed that the high menin expression was positively correlated with the age of patients, tumor stage, and lymph node metastasis (Table 1). As shown in Figure 1D, the representative immunohistochemistry staining images were presented. The other clinical characteristics, including sex and smoking history, were not directly associated with the expression of menin (Table 1). Then, we also investigated the biological role of *MEN1* in BLCA using Gene Set Enrichment Analysis (GSEA) based on mRNA expression data from the TCGA database, which showed that high levels of *MEN1* were positively correlated with cell cycle-related genetic features (Fig. 1E), including cyclin-dependent kinase 1 (*CDK1*), cyclin-dependent kinase 2 (*CDK2*), cyclin-dependent kinase 4 (*CDK4*), cyclin A2 (*CCNA2*), cyclin B1 (*CCNB1*), and cyclin E1 (*CCNE1*) (Fig. 1F).

**Figure 1:**
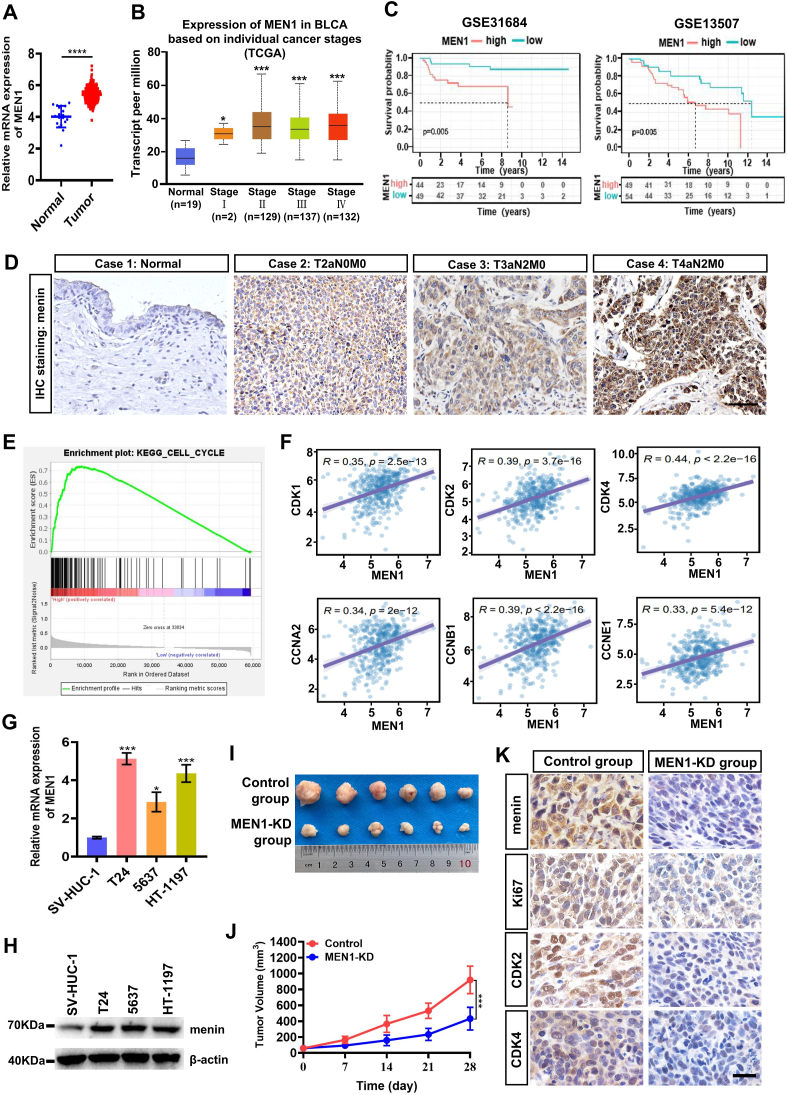
The *MEN1* gene is identified as an oncogene in BLCA. **(A)** The expression status of the *MEN1* gene in BLCA datasets based on the TCGA database. **(B)** The correlation between the *MEN1* gene and tumor stages in BLCA tissues based on the TCGA database. **(C)** The survival analysis indicates that the *MEN1* gene is a risk factor and is related to the survival and prognosis of BLCA tissues based on the GEO database (GSE31684 and GSE13507). **(D)** Representative immunohistochemistry staining images are presented for immunostaining of menin in different stages of BLCA tissues. Scale bar, 100 μm. **(E)** Enrichment analysis of the high expression of the *MEN1* gene of BLCA patients based on GSEA 4.2.3 software (https://www.gsea-msigdb.org/gsea/index.jsp). **(F)** The correlation between the *MEN1* gene and cell cycle checkpoint-related markers in BLCA based on the TCGA database. **(G, H)** The quantitative real-time PCR and western blotting assays analyzed the expression level of the *MEN1* gene and menin protein in human immortalized uroepithelial cell line (SV-HUC-1) and BLCA cell lines (T24, 5637, and HT-1197), respectively. **(I)** Images of representative nude mouse xenograft model studies. T24 tumor xenografts excised from male BALB/c (nu/nu) nude mice after 28 days of treatments with the *in vivo* RNA interfering of *MEN1* knockdown (MEN1-KD group) or control. **(J)** Days versus tumor volume curves for T24 tumor xenografts showed that MEN1-KD inhibited tumor growth compared with the control group. **(K)** Representative immunohistochemistry staining images of menin, Ki67, CDK2, and CDK4 were presented in MEN1-KD-treated or control-treated T24 xenograft nude mice tissues. Scale bar, 50 μm ns, non-significant; ∗*p* < 0.05, ∗∗*p* < 0.01, ∗∗∗*p* < 0.001, and ∗∗∗∗*p* < 0.001 versus control. MEN1, multiple endocrine neoplasia type 1; BLCA, bladder cancer; CDK2/4, cyclin-dependent kinase 2/4.

**Table 1:** Association between menin expression in tumor tissues and clinicopathological characteristics in patients with BLCA (*n* = 90).

Variable	Number of cases (%)	menin		*p*-value
		High (%)	Low (%)	
Gender				0.8037
Male (%)	76 (84.4)	58(82.9)	18(90.0)	
Female (%)	14 (15.6)	12(17.1)	2(10.0)	
Age(years)	62.3 ± 11.21	64.6 ± 8.76	54.4 ± 15.00	0.0002
Tumor TNM				0.0001
Normal, T1, T2 (%)	62 (68.9)	42 (60.0)	20 (100.0)	
T3-T4 (%)	28 (31.1)	28 (40.0)	0 (0)	
Tumor stage				
Normal (%)	5 (5.6)	0 (0)	5 (25.0)	
Stage 1 (%)	32 (35.5)	20 (28.6)	12 (60.0)	0.0131
Stage 2 (%)	18 (20.0)	15 (21.4)	3 (15.0)	0.035
Stage 3 (%)	35 (38.9)	35 (50.0)	0 (0)	0.0118
Lymph node metastasis				0.0005
Yes (%)	17 (18.9)	17 (24.3)	0 (0)	
No (%)	73 (81.1)	53 (75.7)	20 (100.0)	
Smoking history				0.826
Yes (%)	54 (60.0)	42 (60.0)	12 (60.0)	
No (%)	36 (40.0)	28 (40.0)	8 (40.0)	

Furthermore, we verified the expression of *MEN1* gene and menin protein in BLCA cell lines. We found a significant up-regulation of menin not only at the mRNA level but at the protein level in T24, 5637, and HT-1197 cells, when compared with SV-HUC-1 cells (Fig. 1G, H). Finally, we studied the biological function of menin in T24 cells xenografted nude mice model *in vivo*. Subsequently, we found that *MEN1* knockdown (MEN1-KD) by RNA interfering *in vivo* system, significantly decreased the size of xenograft tumors in nude mice model (Fig. 1I, J). We also observed that MEN1-KD induced a strong reduction of the menin, Ki67, CDK2, and CDK4 expression in the xenografted tumor tissues (Fig. 1K), suggesting that menin is an oncogenic factor in BLCA cells *in vivo*.

### Menin promotes the proliferation of BLCA cells via modulating cell cycle transition

To assess the function of the *MEN1* gene in BLCA cells, we performed the RNA interfering (siMEN1: siMEN1#1 or siMEN1#2) to MEN1-KD in BLCA cells, with non-targeting siRNA as negative control (siCtrl). First, to determine the efficiency of MEN1-KD upon the treatment of siMEN1#1 and siMEN1#2 in BLCA cells, we analyzed the *MEN1* mRNA and menin protein expression levels in siMEN1#1 or siMEN1#2-treated cancer cells by RT-qPCR and western blotting assay. As shown in Figure 2A and B, the *MEN1* gene or menin protein was significantly reduced in MEN1-KD T24, 5637, and HT-1197 cells compared with siCtrl-treated cancer cells at 72 h post-transfection. We then performed the CCK-8 assay and colony formation assay to analyze the cell proliferation in MEN1-KD- or siCtrl-treated T24, 5637, and HT-1197 cells. Subsequently, the proliferation of T24, 5637, and HT-1197 cells was significantly inhibited after MEN1-KD treatment (Fig. 2C), and the results of the colony formation assay further confirmed this observation (Fig. 2D). Furthermore, we also demonstrated that MEN1-KD induced cell cycle arrest at G1/S phase in BLCA cells (Fig. 2E). These results indicate that menin seems to be a pivotal regulator in regulating cell cycle transition in BLCA cells. We further verified that MEN1-KD caused the strong reduction of factors' expression involved in cell cycle transition, including CDK2, CDK4, cyclin D1 (CCND1), and CCNE1 in BLCA cells (Fig. 2F, G). Collectively, our findings demonstrate, to our knowledge for the first time, that menin promotes proliferation via enhancing cell cycle transition in BLCA cells.

**Figure 2:**
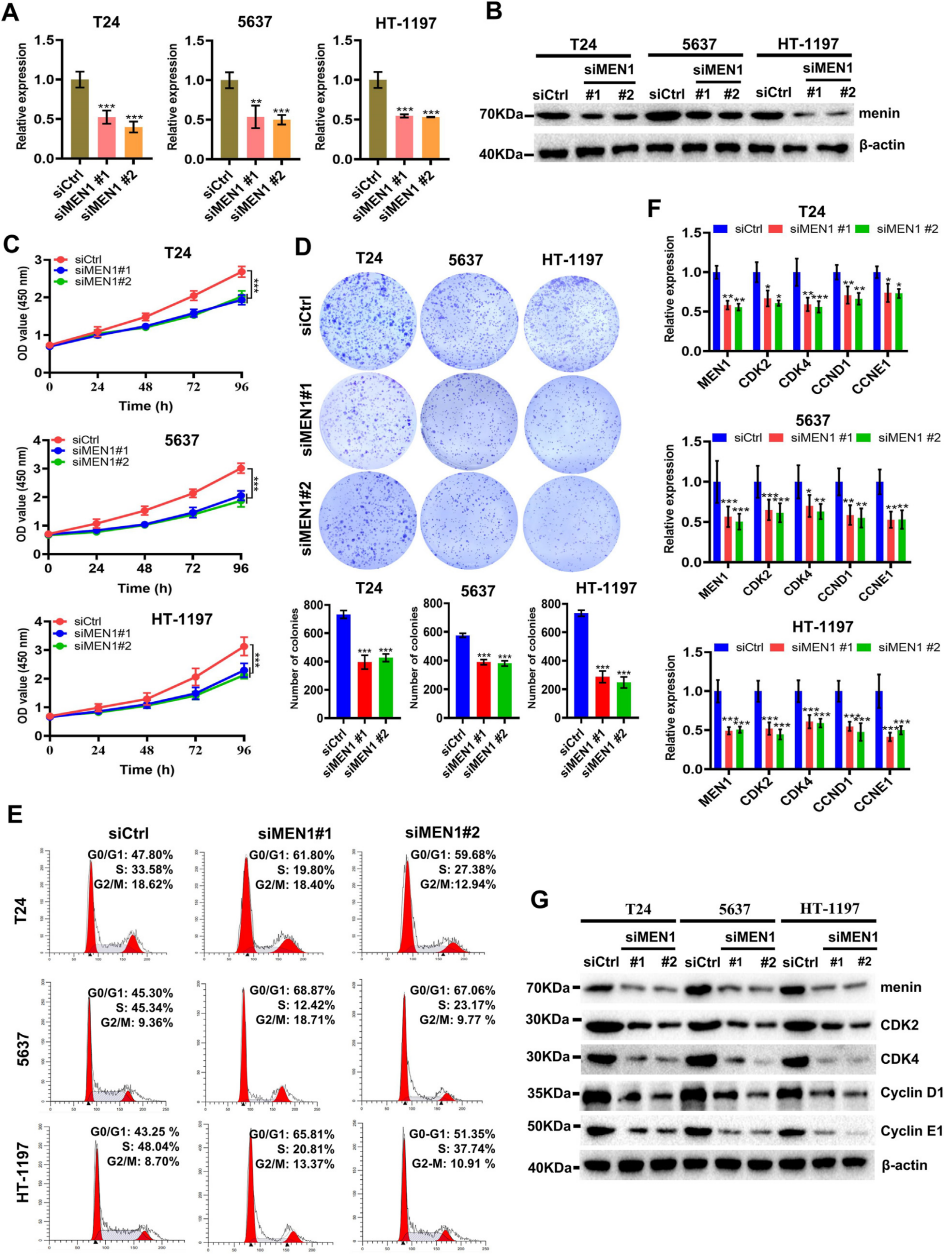
Reduced menin inhibits BLCA cell proliferation via modulating cell cycle transition. **(A)** RT-qPCR analysis of *MEN1* transcription in siCtrl-, siMEN1#1-, or siMEN1#2-treated T24, 5637, and HT-1197 BLCA cells. Data were normalized against ACTB and represented as fold change. **(B)** Western blotting analysis showed menin expression in siCtrl-, siMEN1#1-, or siMEN1#2-treated T24, 5637, and HT-1197 BLCA cells. **(C)** CCK-8 analysis showed the effect of MEN1-KD (siMEN1#1, siMEN1#2) on the cell proliferation of the tested BLCA cell lines. **(D)** Representative images of colony formation assays and their quantification in MEN1-KD T24, 5637, and HT-1197 cells. **(E)** MEN1-KD induces cell cycle arrest at the G1/S phase in T24, 5637, and HT-1197 cells, compared with the cells transfected with siCtrl-by flow cytometry. Percentages (%) of cell populations at different stages of cell cycles are listed within the panels. **(F, G)** The relative expression of *MEN1*, *CDK2*, *CDK4*, *CCND1*, and *CCNE1* were measured in MEN1-KD T24, 5637, and HT-1197 cells by RT-qPCR or western blotting analysis. The experiment was repeated three times, and representative results are presented. ns, non-significant; ∗*p* < 0.05, ∗∗*p* < 0.01, and ∗∗∗*p* < 0.001 versus control. BLCA, bladder cancer; RT-qPCR, quantitative real-time PCR; MEN1, multiple endocrine neoplasia type 1; CDK2/4, cyclin dependent kinase 2/4; CCND1, cyclin D1; CCNE1, cyclin E1.

### MEN1-KD reduces β-catenin expression in BLCA cells

To study the mechanism of menin regulating the proliferation in BLCA cells, we performed RNA sequencing analysis across the entire genome. The total RNA of T24 cells transfected with a control (siCtrl) or siMEN1 (siMEN1#1 + siMEN1#2) was isolated and sequenced. A total of 7200 genes were differentially expressed upon MEN1-KD treatment (Fig. 3A). Then, the Kyoto Encyclopedia of Genes and Genomes (KEGG) pathway analysis was used to examine the functions of the differentially expressed genes. The top 10 results for each functional group are presented in Figure 3B. KEGG pathway enrichment analysis linked differentially expressed genes to processes involving the Wnt signaling pathway, cell cycle, autophagy, tumor necrosis factor signaling pathway, mitophagy, polycomb repressive complex, nucleotide excision repair, DNA replication, apoptosis-multiple species, and RNA polymerase.

**Figure 3:**
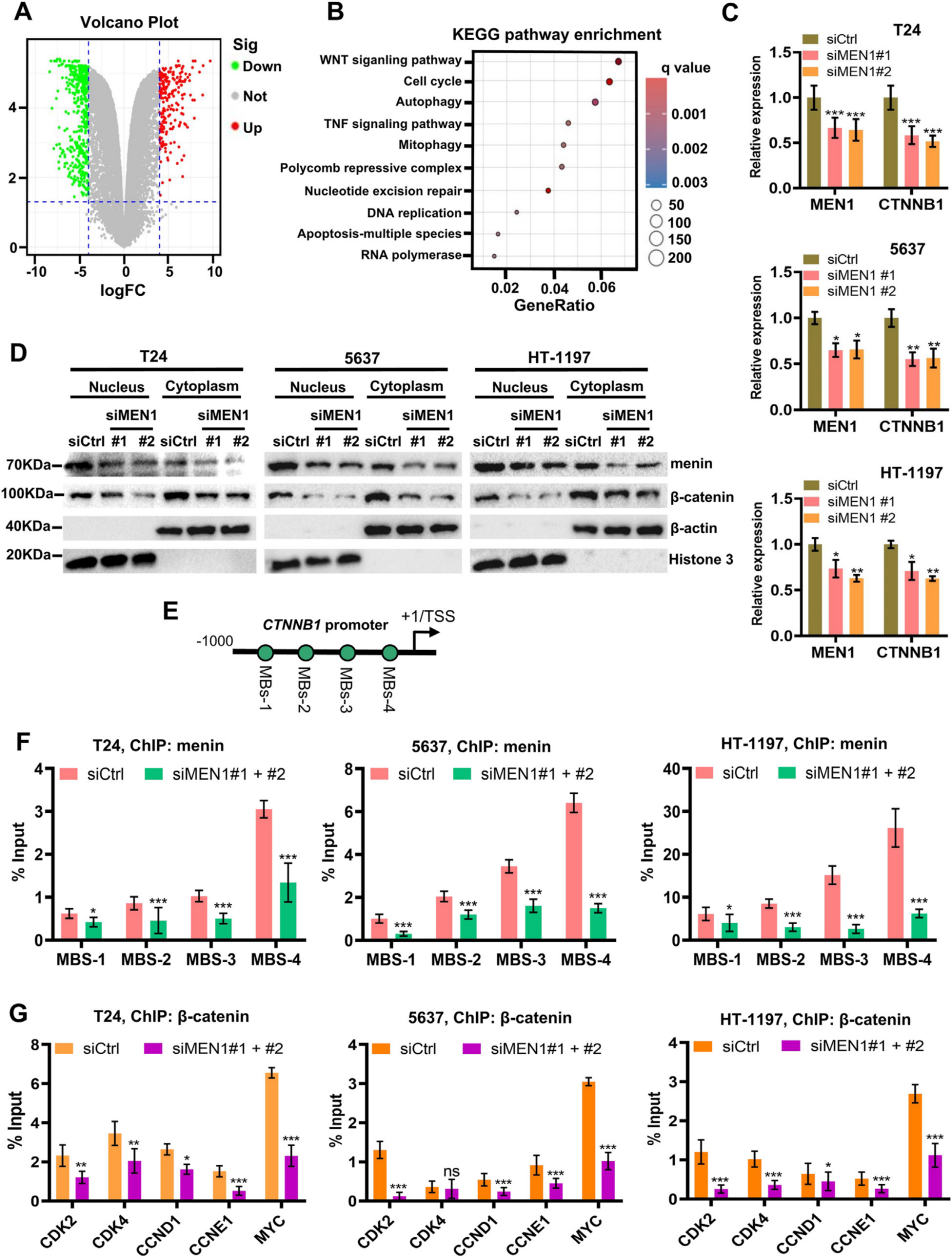
*MEN1* silencing reduces cell growth via suppressing Wnt/β-catenin signaling in BLCA cells. **(A)** T24 cells transfected by siCtrl or siMEN1-#1 + #2 were harvested for transcriptomic RNA sequencing. The volcano plot depicts the gene expression changes in siMEN1-treated T24 cells. **(B)** KEGG pathway enrichment analysis based on RNA sequencing showed the pathway enrichment of differentially expressed genes. The bubble size indicates the number of genes. The color bar indicates the corrected *p*-value. The threshold for differential was set at 2-fold change, and *p* < 0.05, as determined by DESeq2. **(C)** RT-qPCR analysis showed the down-regulated *CTNNB1* expression in MEN1-KD T24, 5637, and HT-1197 cells. **(D)** The protein expression of β-catenin was analyzed by western blotting in MEN1-KD T24, 5637, and HT-1197 cells, as indicated. **(E)** Schematic representation of the position of ChIP-qPCR primers along the *CTNNB1* promoter. **(F)** The levels of menin recruitment to the *CTNNB1* promoter in siCtrl- or siMEN1#1 + #2-transfected BLCA cells, detected by ChIP-qPCR assay. **(G)** The β-catenin recruitment on the *CDK2*, *CDK4*, *CCNE1*, *CCND1*, and *MYC* promoter in siCtrl- or siMEN1#1 + #2-transfected BLCA cells, detected by ChIP-qPCR. ns, non-significant; ∗*p* < 0.05, ∗∗*p* < 0.01, and ∗∗∗*p* < 0.001 versus control. BLCA, bladder cancer; RT-qPCR, quantitative real-time PCR; MEN1, multiple endocrine neoplasia type 1; CTNNB1, catenin beta 1; ChIP, chromatin immunoprecipitation; CDK2/4, cyclin dependent kinase 2/4; CCND1, cyclin D1; CCNE1, cyclin E1.

The molecular mechanisms of tumor initiation and progression are related to the activation of the Wnt signaling pathway.^26^ This pathway facilitates the elevation of β-catenin levels, promoting its translocation to the nucleus, where it interacts with transcription factors in the TCF/LEF family, thereby establishing a common pathway for Wnt signal activation.^27^ The β-catenin-TCF/LEF complex regulates cell proliferation by inducing expression of downstream target genes.^28^ In addition, β-catenin (gene name: *CTNNB1*) is an important tumor promoter in BLCA.^29^ More intriguingly, menin has been reported to interact with β-catenin to promote tumorigenesis *in vivo*^30^ and *in vitro*.^24^ However, the crosstalk between menin and β-catenin in BLCA cells is still unknown. Intriguingly, we found MEN1-KD significantly down-regulated the expression of β-catenin at mRNA and protein levels in T24, 5637, and HT-1197 cells (Fig. 3C, D). To further study the *CTNNB1* gene regulation by menin in BLCA cells, we sought to determine the occupancy of menin on the *CTNNB1* promoter by ChIP analysis. Subsequently, we demonstrated menin bound to the proximal promoter of the *CTNNB1* gene encompassing the −1000 to −10 region flanking its transcription start site, with the menin enrichment signal diminishing drastically when cells were treated with siMEN1 (Fig. 3E, F). Moreover, we also demonstrated that *MEN1* silencing resulted in decreasing the binding of β-catenin to the promoter of its downstream target genes, including *CDK2*, *CDK4*, *CCND1*, *CCNE1*, or *MYC* (Fig. 3G). Our data revealed that menin could regulate *CTNNB1* transcription via binding to the *CTNNB1* proximal promoter and subsequently trigger Wnt/β-catenin signaling in BLCA cells.

### Menin regulates the *TFAP2C* transcription to promote the proliferation of BLCA cells

We then compared the gene expression patterns of siCtrl-treated cells to those of MEN1-KD BLCA cells to identify genes potentially regulated by *MEN1*. The transcription factor AP-2 gamma (*TFAP2C*) gene was most significantly down-regulated in MEN1-KD T24 cells among the top 20 down-regulated genes, based on our RNA sequencing data (Fig. 4A). TFAP2C plays an important role in regulating cell proliferation, cell cycle progression, and apoptosis, and participates in the development of several cancers and influences tumor sensitivity to chemotherapy.^31^, ^32^, ^33^ More recently, studies have demonstrated that TFAP2C promotes cisplatin resistance *in vivo* and *in vitro* by inducing the levels of EGFR and NF-κB activation in BLCA cells.^34^ We then validated the function of TFAP2C in BLCA cells, we knocked down the *TFAP2C* gene (TFAP2C-KD) expression in BLCA cells by two different RNA interfering (siTFAP2C #1 and siTFAP2C #2) (Fig. 4B, C). As shown in Figure 4D and E, TFAP2C-KD significantly reduced the proliferation of T24, 5637, and HT-1197 cells indicated by CCK-8 and cell colony formation assay. These results are consistent with the previous report.^34^ However, it remains unclear whether menin promotes the growth of BLCA cells by regulating TFAP2C expression. Intriguingly, we found that MEN1-KD caused a reduction of TFAP2C expression at mRNA and protein levels in T24, 5637, and HT-1197 cells (Fig. 4F, G). We also found that MEN1-KD reduced the TFAP2C expression in the T24 cells-xenografted nude mice model (Fig. 4H).

**Figure 4:**
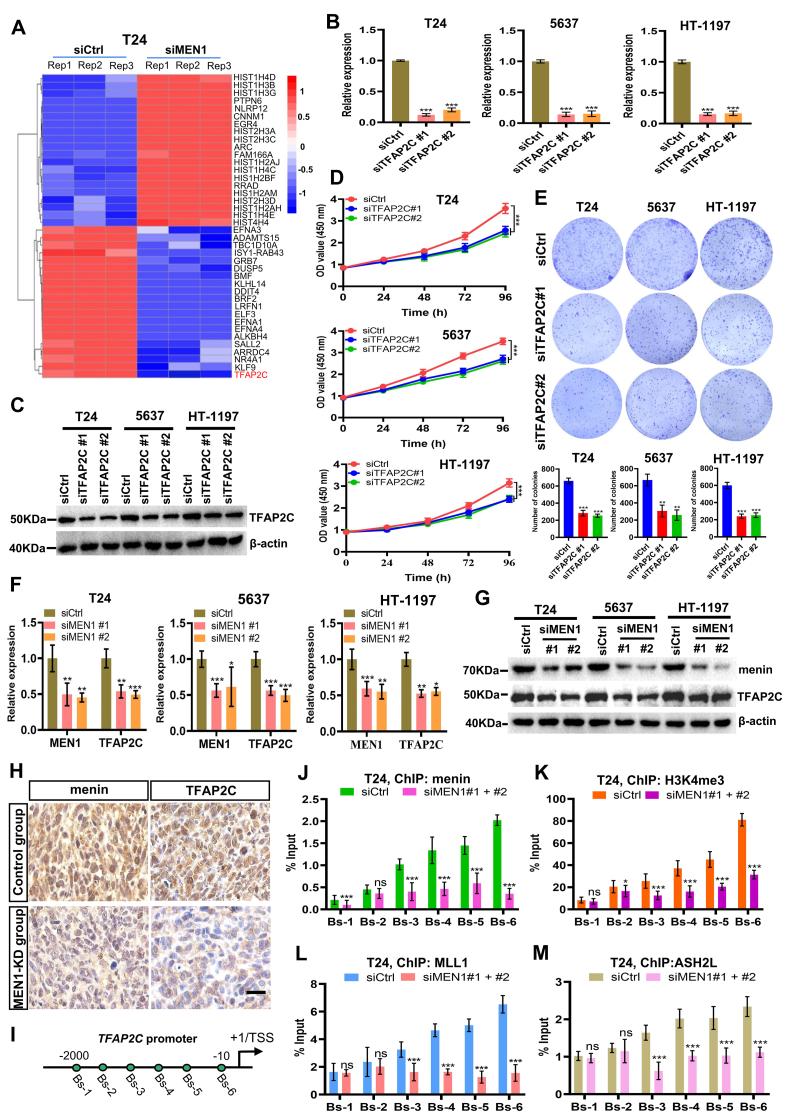
Menin regulates *TFAP2C* transcription to promote BLCA cell growth. **(A)** Heatmap of transcriptional changes triggered by negative control or MEN1-KD based on RNA sequencing data of T24 cells transfected with siCtrl or siMEN1. **(B, C)** mRNA and protein expression of TFAP2C was analyzed by RT-qPCR and western blotting in TFAP2C-KD T24, 5637, and HT-1197 cells. **(D)** CCK-8 analysis showed the effect of TFAP2C-KD (siTFAP2C#1, siTFAP2C#2) on the cell proliferation of the tested BLCA cell lines. **(E)** Representative images of colony formation assays and their quantification in TFAP2C-KD T24, 5637, and HT-1197 cells. **(F)** RT-qPCR analysis showed the down-regulated TFAP2C expression in MEN1-KD T24, 5637, and HT-1197 cells. **(G)** Western blotting analysis showed MEN1-KD caused a strong reduction of TFAP2C expression in T24, 5637, and HT-1197 cells. **(H)** Representative immunohistochemistry images were presented for the immunostaining of menin and TFAP2C in MEN1-KD-treated or negative control-treated T24 xenograft nude mouse tissues. **(I)** Schematic representation of the position of ChIP-qPCR primers along the *TFAP2C* promoter. **(J**–**M)** The levels of menin, H3K4me3, MLL1, or ASH2L recruitment to the *TFAP2C* promoter in siCtrl- or siMEN1#1 + #2-transfected T24 cells, detected by ChIP-qPCR. Scale bar, 50 μm ns, non-significant; ∗*p* < 0.05, ∗∗*p* < 0.01, and ∗∗∗*p* < 0.001 versus control. TFAP2C, transcription factor AP-2 gamma; BLCA, bladder cancer; RT-qPCR, quantitative real-time PCR; MEN1, multiple endocrine neoplasia type 1; ChIP, chromatin immunoprecipitation; H3K4me3, histone 3 lysine 4 trimethylation; MLL1, mixed lineage leukemia 1.

To further investigate the regulation of TFAP2C by menin, as indicated by the observations in BLCA cells, we conducted ChIP assays to determine the presence of menin on the *TFAP2C* promoter. Our results revealed a significant enrichment of menin in the proximal region of the *TFAP2C* promoter, specifically between −2000 and −10 bp surrounding the transcription start site (Fig. 4I, J). This menin occupancy signal decreased significantly when cells were treated with siMEN1 (Fig. 4J; Fig. S2A). These results indicate that TFAP2C seems to be a new downstream target of menin in BLCA cells. Additionally, we found that this region was highly methylated at histone 3 lysine 4 trimethylation (H3K4me3) (Fig. 4K; Fig. S2B) and was co-occupied by the COMPASS-like components MLL1 (Fig. 4L; Fig. S2C) and ASH2L (Fig. 4M; Fig. S2D), suggesting that the complex binds to this specific region and plays a role in TFAP2C regulation. Our data thus demonstrate that menin regulates the transcription of the *TFAP2C* gene via binding to the *TFAP2C* promoter region, medicated by the MLL complex in BLCA cells.

### TFAP2C is essential for the regulation of *CTNNB1* by menin in BLCA cells

**Figure 5:**
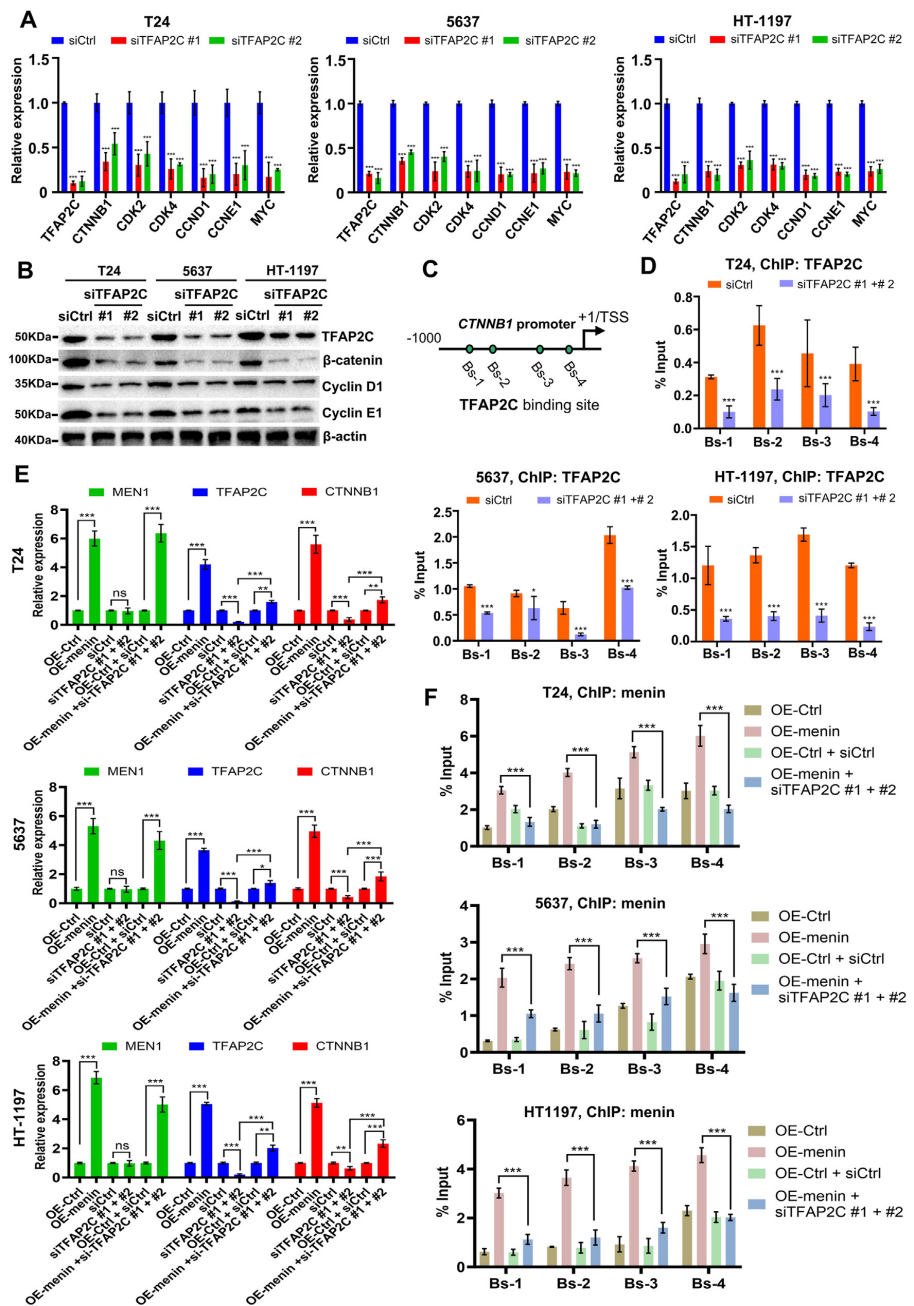
TFAP2C is crucial for the regulation of *CTNNB1* by menin in BLCA cells. **(A, B)** mRNA and protein expression of *TFAP2C*, *CTNNB1*, *CDK2*, *CDK4*, *CCND1*, *CCNE1*, and *MYC* were analyzed by RT-qPCR and western blotting in TFAP2C-KD T24, 5637, and HT-1197 cells. **(C)** Schematic representation of the position of ChIP-qPCR primers along the *CTNNB1* promoter. **(D)** The levels of TFAP2C recruitment to the *CTNNB1* promoter in siCtrl or siTFAP2C-treated T24, 5637, and HT-1197 cells, detected by ChIP-qPCR. **(E)** RT-qPCR analysis showed the expression of *MEN1*, *TFAP2C*, and *CTNNB1* in OE-menin-treated, siTFAP2C-treated, or OE-menin plus siTFAP2C-treated BLCA cells. **(F)** The levels of menin recruitment to the *CTNNB1* promoter in OE-menin-treated, or OE-menin plus siTFAP2C-treated T24, 5637, and HT-1197 cells, detected by ChIP-qPCR. ns, non-significant; ∗*p* < 0.05, ∗∗*p* < 0.01, and ∗∗∗*p* < 0.001 versus control. TFAP2C, transcription factor AP-2 gamma; BLCA, bladder cancer; RT-qPCR, quantitative real-time PCR; MEN1, multiple endocrine neoplasia type 1; CTNNB1, catenin beta 1; ChIP, chromatin immunoprecipitation; CDK2/4, cyclin dependent kinase 2/4; CCND1, cyclin D1; CCNE1, cyclin E1.

Among the TFAP2 family members, TFAP2A, TFAP2B, and TFAP2E interact with β-catenin to inhibit or promote oncogenesis in different types of cancers.^35^ However, the relationship between TFAP2C and β-catenin in BLCA cells remains elusive. Intriguingly, we found that TFAP2C-KD significantly reduced the mRNA expression of *CTNNB1* and its downstream targets' mRNA expression, such as *CDK2*, *CDK4*, *CCND1*, *CCNE1*, and *MYC* (Fig. 5A). Moreover, the protein expression of β-catenin, CCND1, and CCNE1 was inhibited in TFAP2C-KD-treated T24, 5637, and HT-1197 cells (Fig. 5B). Furthermore, *CTNNB1* knockdown did not affect the TFAP2C expression at the mRNA or protein level in these cancer cells (Fig. S3). We then studied the mechanism of the activation of β-catenin by TFAP2C in BLCA cells. ChIP-qPCR results showed that TFAP2C bound to the *CTNNB1* proximal promoter, subsequently regulating the *CTNNB1* gene transcription (Fig. 5C, D). Moreover, we found that TFAP2C-KD significantly reduced the enrichment of β-catenin on the *CCND1*, *CCNE1*, and *MYC* promoter (Fig. S4). These results indicate that TFAP2C is a new regulator in triggering the transcription of the *CTNNB1* gene, especially in BLCA cells.

We next sought to study whether TFAP2C participated in the regulation of *CTNNB1* by menin in BLCA cells. We analyzed the *MEN1*, *TFAP2C*, and *CTNNB1* expression in menin over-expressed T24, 5637, and HT-1197 cells. The plasmid of menin overexpression (OE-menin) was described in our previous study.^24^ As shown in Figure 5E, OE-menin significantly induced the expression of *TFAP2C* and *CTNNB1* in these BLCA cells. We also observed that although TFAP2C-KD reduced the *CTNNB1* expression, TFAP2C-KD did not affect the expression of the *MEN1* gene. More intriguingly, we found that TFAP2C-KD could still reduce the *CTNNB1* expression even upon OE-menin treatment in T24, 5637, and HT-1197 cells. The results indicated that TFAP2C-KD could reverse the activation of *CTNNB1* by menin in BLCA cells.

Then, we explored the mechanism of menin and TFAP2C co-regulating *CTNNB1* transcription in BLCA cells. ChIP-qPCR results showed that although OE-menin increased menin enrichment on the *CTNNB1* promoter, TFAP2C-KD caused a significant reduction of menin binding to the *CTNNB1* promoter in T24, 5637, and HT-1197 cells (Fig. 5F). These results indicate that TFAP2C is required for the regulation of *CTNNB1* transcription by menin in BLCA cells.

### Small molecule inhibitor, BAY-155, inhibits BLCA cell proliferation by modulating the TFAP2C/β-catenin axis

**Figure 6:**
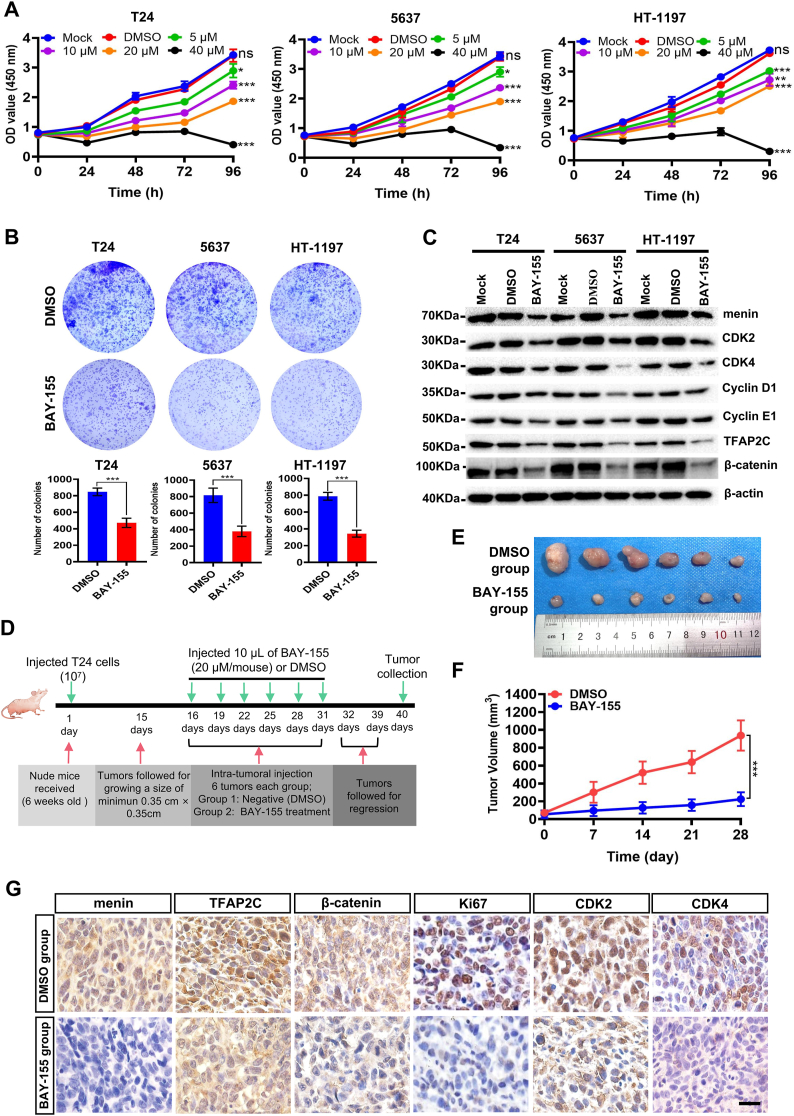
BAY-155 inhibits the proliferation of bladder cancer cells. **(A)** CCK-8 analysis showed the effect on the cell proliferation of menin inhibitor BAY-155 with different doses as indicated in T24, 5637, and HT-1197 cells. **(B)** Representative images of foci formation assays and their quantification in BAY-155-treated T24, 5637, and HT-1197 cells. **(C)** The menin, CDK2, CDK4, CCND1, CCNE1, TFAP2C, and β-catenin expression were evaluated by western blotting analysis in T24, 5637, and HT-1197 cells treated by menin inhibitor BAY-155. **(D)** Schematic diagram of the strategy used for evaluating the effect of BAY-155 on T24 cell growth by xenografts in mice. **(E)** Photograph and comparison of excised xenograft tumor size in BAY-155- or DMSO-treated T24 cells. **(F)** The tumor volume of xenograft nude mice treated with DMSO or BAY-155 was measured. **(G)** Representative immunohistochemistry staining images of menin, TFAP2C, β-catenin, Ki67, CDK2, and CDK4 were presented in BAY-155- or DMSO-treated T24 cell xenograft nude mouse tissues. Scale bar, 50 μm ns, non-significant; ∗*p* < 0.05, ∗∗*p* < 0.01, and ∗∗∗*p* < 0.001 versus control. TFAP2C, transcription factor AP-2 gamma; CDK2/4, cyclin dependent kinase 2/4; CCND1, cyclin D1; CCNE1, cyclin E1.

Accumulating studies have demonstrated that menin small molecule inhibitors (MIs) are a class of therapeutic agents that show promise in the treatment of various cancers. Brzezinka et al reported a type of MIs, BAY-155, displayed a promising anti-proliferative activity in BLCA cells.^22^ In the current study, we further investigated the function and underlying mechanisms of BAY-155 in BLCA cells. As shown in Figure 6A and B, compared with DMSO treatment, BAY-155 displayed a dose-dependent inhibiting effect in T24, 5637, and HT-1197 cells by CCK8 and foci formation analysis. Intriguingly, we also found that the expression of menin, TFAP2C, and β-catenin were obviously decreased in BAY-155-treated BLCA cells (Fig. 6C). Furthermore, BAY-155 inhibited the expression of factors associated with the cell cycle in these cancer cells (Fig. 6C). These results suggest that BAY-155 suppresses the cell proliferation of BLCA cells most likely via inhibiting the menin/TFAP2C/β-catenin signaling axis.

Moreover, we then verified the effects of BAY-155 on the growth capabilities of BLCA cells *in vivo*. We applied DMSO- (control) or BAY-155 treatment to the nude mice model following the described strategy in Figure 6D. As shown in Figure 6E and F, the average size of tumors derived from BAY-155-treated mice was several folds smaller compared with the tumors derived from DMSO-treated mice. Immunostaining assay analyzed the expression of menin, TFAP2C, β-catenin, Ki67, CDK2, and CDK4, which were significantly reduced in the xenograft tissues upon BAY-155 treatment, compared with the control DMSO group (Fig. 6G). Meanwhile, hematoxylin-eosin staining analysis showed that BAY-155 treatment did not cause damage to the heart, liver, kidneys, and lungs in xenografted nude mice (Fig. S5). These results indicate that BAY-155 suppresses the BLCA tumor growth by targeting the menin/TFAP2C/β-catenin signaling axis both *in vitro* and *in vivo*.

## Discussion

The past several decades have seen a rapid expansion in understanding the function of menin in cell growth and cancer progression. While previous studies have suggested that the regulation of genes by menin seems no pattern, recent evidence indicates that the models for menin regulation could be context-dependent: menin serves both as a tumor suppressor^36^ and oncoprotein,^37^ depending on different circumstances. However, the regulatory and functional role of menin during tumorigenesis in BLCA is still unknown. In the current study, we report that menin acts as a tumor promoter and is positively correlated with poor survival of BLCA. Menin coordinates with TFAP2C to elevate β-catenin expression and impoverishes the proliferation of BLCA cells by triggering the Wnt signaling pathway.

Being well-established as a cancer-promoting molecule, menin is overexpressed in several cancers, which indicates a poor prognosis of leukemia, liver cancer, or prostate cancer patients. Consistently, we here uncovered a previously unknown oncogenic role for menin in BLCA. We found that menin was up-regulated in BLCA tissues and associated with the poor prognosis of BLCA patients. The cell cycle checkpoint is closely related to BLCA prognosis.^38^ Several studies have demonstrated that menin is a pivotal regulator of the cell cycle,^39^ and acts as a potential therapeutic target for cancers. We demonstrated that MEN1-KD inhibited BLCA cell growth *in vitro* and *in vivo* by altering the G1/S transition.

Furthermore, the current RNA sequencing analysis also reveals that menin promotes the cell cycle transition of BLCA cells via targeting the Wnt/β-catenin pathway. The wnt/β-catenin signaling pathway plays a major impact on proliferation during cancer progression.^40^ β-catenin is the central regulator in this signaling pathway.^41^ Once the Wnt signaling pathway is activated, which causes the degradation of β-catenin, is inhibited, and β-catenin enters into the nucleus to promote the expression of downstream target genes such as c-Myc and CCND1, and finally promote the malignant progression of cells.^42^ Importantly, it has also been suggested that β-catenin could play a critical role in the proliferation of BLCA.^29^ Intriguingly, our previous work has demonstrated that menin interacts with β-catenin and controls the binding of β-catenin to the *MYC* promoter in prostate cancer cells.^25^ Moreover, studies also reported that *Men1* deficiency leads to nuclear translocation and activation of β-catenin in mouse insulinoma.^43^ However, there is no study exploring the association between menin and Wnt/β-catenin signaling pathway in BLCA yet. Here, our work provides compelling evidence, to our knowledge for the first time, that menin critically regulates *CTNNB1* transcription, likely through its binding to the proximal *CTNNB1* promoter, mediated by H3K4me3. *MEN1* silencing results in decreased expression of known β-catenin target genes and enhanced cell cycling alterations.

More intriguingly, our RNA sequencing results revealed that MEN1-KD induced a reduction of *TFAP2C* mRNA expression in BLCA cells. The involvement of menin in the regulation of TFAP2C has not been previously addressed before. Our data demonstrated that menin up-regulated the TFAP2C expression via binding to the *TFAP2C* proximal promoter in BLCA cells, mediated by the MLL complex. These results suggest that TFAP2C is a new downstream target for menin in BLCA cells. Importantly, we demonstrate that TFAP2C is required for menin to regulate the *CTNNB1* gene transcription in BLCA cells. TFAP2C, a proliferation-associated transcription factor, is critically involved in the regulation of β-catenin by menin in BLCA cells. It has been documented that activated TFAP2C is critical for the progression and drug resistance of BLCA.^34^ Intriguingly, previous studies have demonstrated that the members of the TFAP2 family, including TFAP2A, TFAP2B, and TFAP2E, inhibit Wnt/β-catenin signaling to limit tumor growth and migration in cervical cancer^44^ and colorectal cancer^45^ cells. However, our current results provide new evidence that TFAP2C promotes proliferation through activating β-catenin expression in BLCA cells. Mechanically, TFAP2C positively regulates *CTNNB1* transcription via binding to the *CTNNB1* promoter in cancer cells. Collectively, distinct effects of TFAP2 on tumor growth, proliferation, and migration are observed in various types of cancer, indicating a complex underlying regulatory network. Therefore, further research is needed to clarify these controversies.

Notably, we also demonstrated that the menin small molecule inhibitor, BAY-155^22^, inhibited the cell proliferation of BLCA cells *in vivo* and *in vitro*. The menin small molecule inhibitors, such as MI-463, MI-503, MI-3454, and VTP-50469, markedly inhibit the proliferation and induce the differentiation of acute leukemia cells and primary patient samples with MLL translocations or NPM1 mutations. In addition, these inhibitors have been attracting attention as a therapeutic method for not only leukemia, but also solid cancers, such as breast cancer, pancreatic cancer, prostate cancer, Ewing sarcoma, and hepatocellular carcinoma.^19^ The cancer-related role of BAY-155 has never been revealed in BLCA before. Here, we demonstrated that BAY-155 not only significantly reduced the expression of menin protein, but suppressed both TFAP2C and β-catenin expression in BLCA cells. These results show that BAY-155 exhibits a strong anti-tumor effect in BLCA via inhibiting the menin/TFAP2C/β-catenin axis.

**Figure 7:**
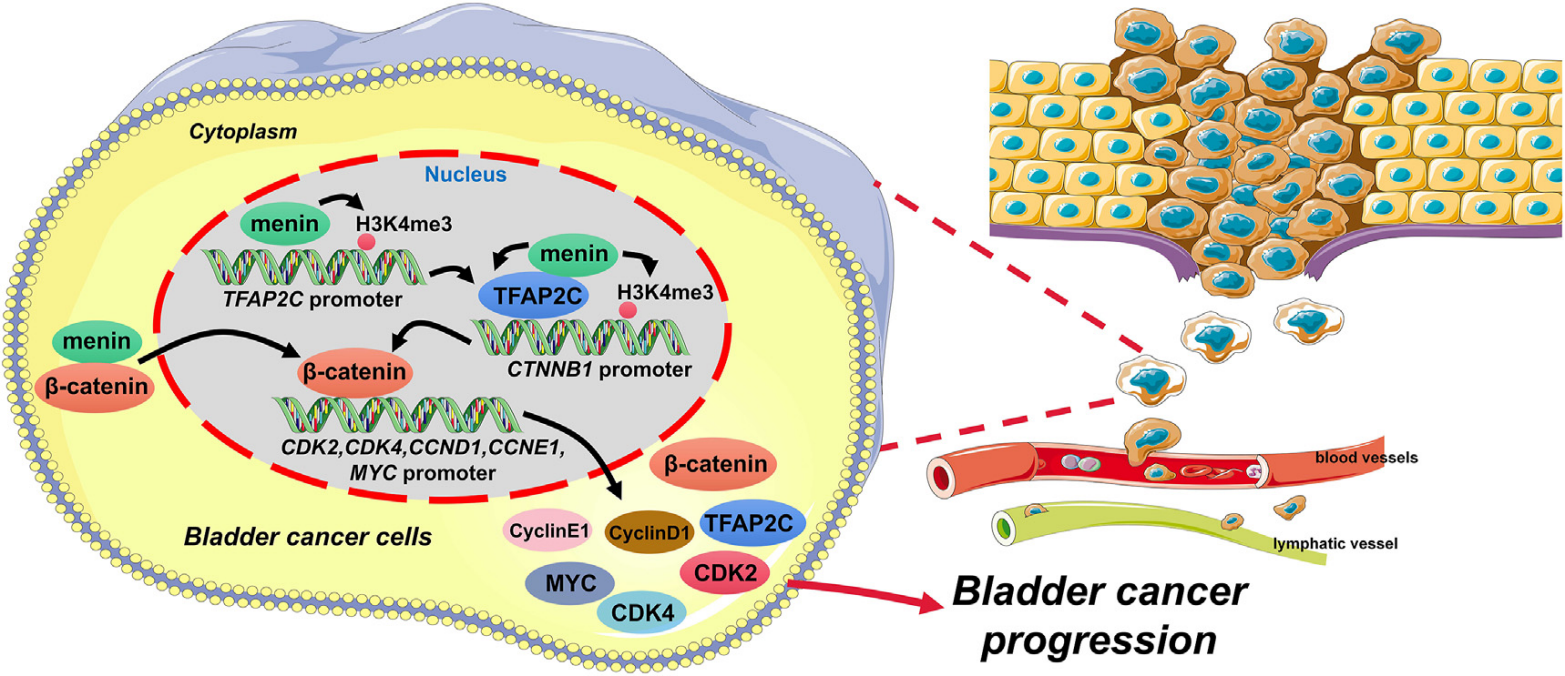
Schematic diagram of the possible mechanism underlying the regulatory effect of menin on the TFAP2C/β-catenin signaling axis in bladder cancer cells. TFAP2C, transcription factor AP-2 gamma.

Collectively, our current study identified that the overexpression of menin facilitated the malignant behaviors of BLCA cells by boosting β-catenin via activating TFAP2C, suggesting that menin might serve as a therapeutic target and prognostic marker for BLCA (Fig. 7).

## CRediT authorship contribution statement

**Qing Shi:** Investigation, Methodology, Validation. **Xiang Pan:** Data curation, Investigation, Methodology, Validation. **Shiheng Zhang:** Data curation, Formal analysis, Resources, Visualization. **Mengyuan Wu:** Formal analysis, Methodology, Validation. **Meiqi Xu:** Methodology, Validation, Visualization. **Yun-Qi Li:** Resources, Writing – review & editing. **Li Zhong:** Resources, Software. **Zi-Qi Wang:** Writing – review & editing. **Wanhai Xu:** Writing – review & editing. **Yakun Luo:** Conceptualization, Funding acquisition, Project administration, Writing – original draft.

## Funding

This work was supported by the 10.13039/501100012166National Key Research and Development Program of China (No. 2022YFA1205704), the 10.13039/501100005046Natural Science Foundation of Heilongjiang Province, China (No. YQ2023H015), the Fundamental Research Funds for the Provincial Universities of Heilongjiang, China (No. 2022KYYWF-0298), the Research Fund of the Fourth Affiliated Hospital of Harbin Medical University (No. HYDSYRCYJ02), the Research Fund of Chongqing Key Laboratory of Development and Utilization of Genuine Medicinal in Three Gorges Reservoir Area (China) (No. KFKT2022011).

## Conflict of interests

The authors declare that they have no known competing financial interests or personal relationships that could have appeared to influence the work reported in this paper.

## Figures and Tables

**figs1:**
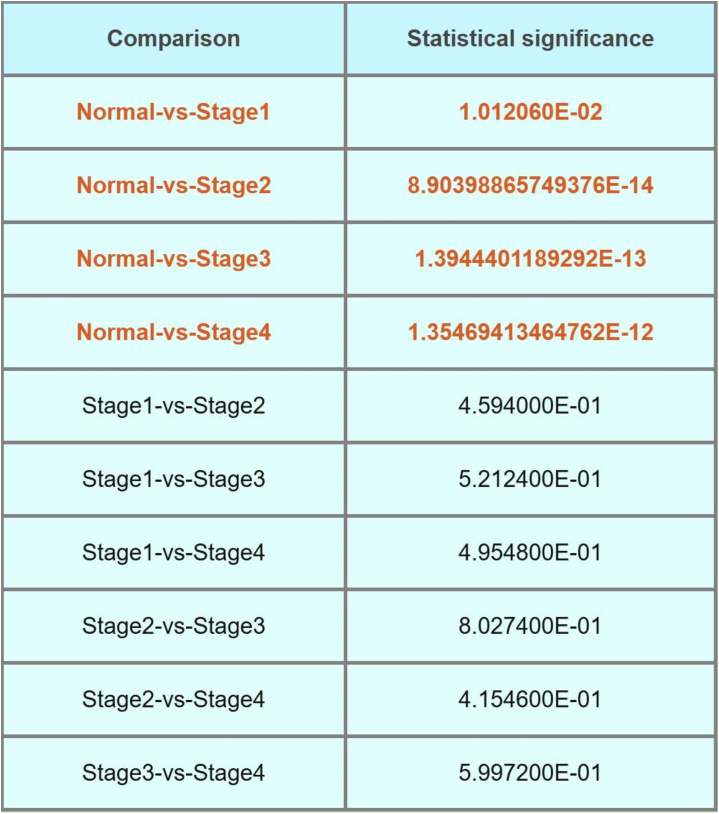


**figs2:**
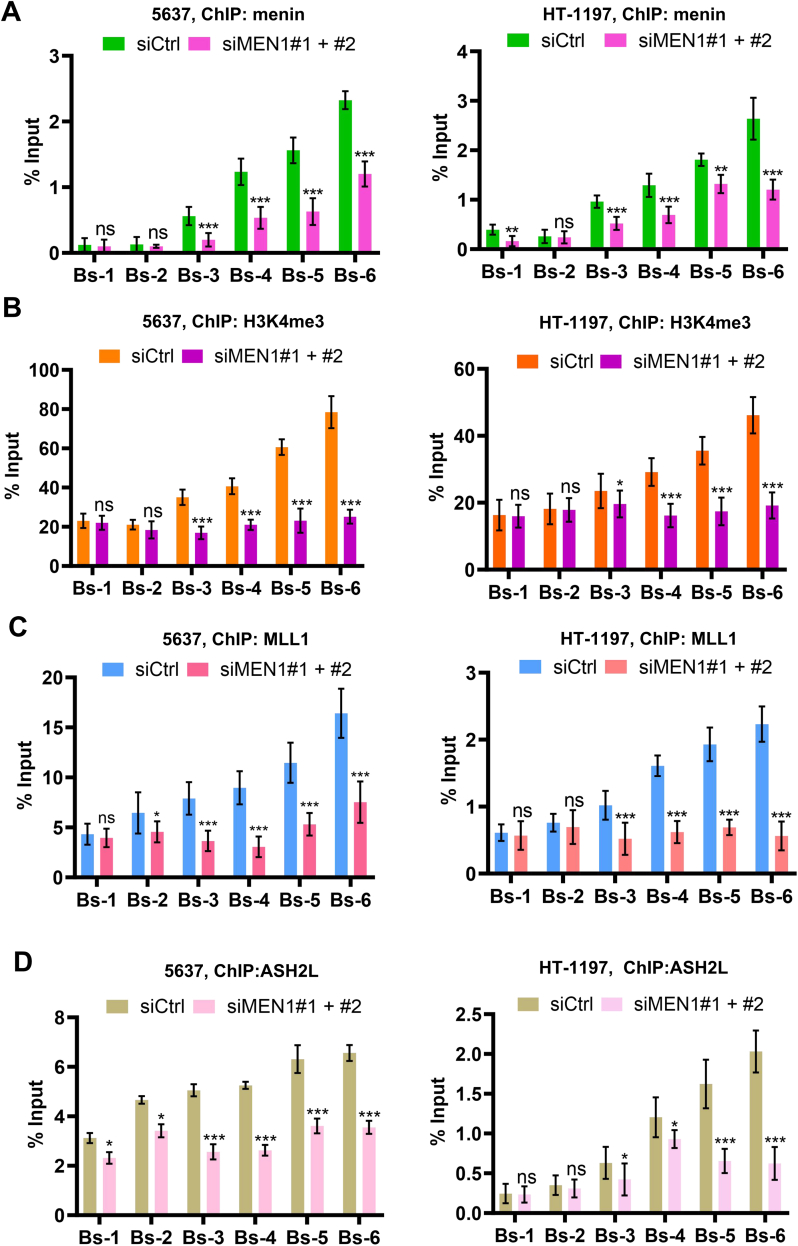


**figs3:**
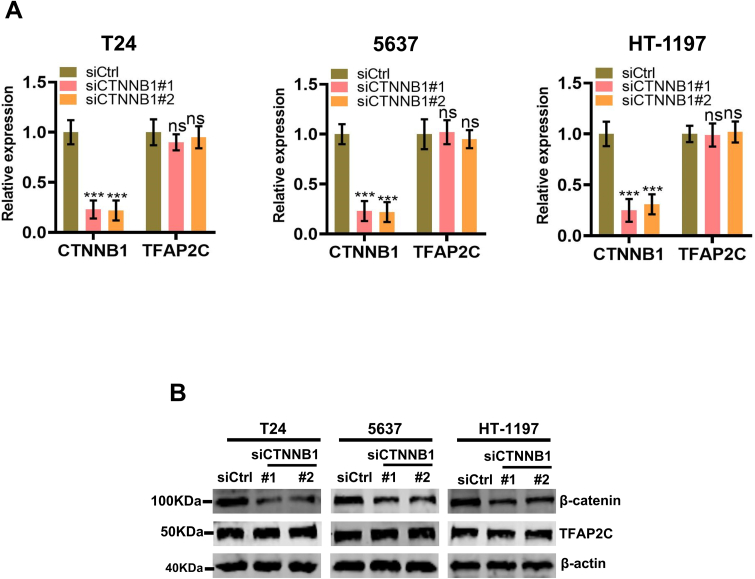


**figs4:**
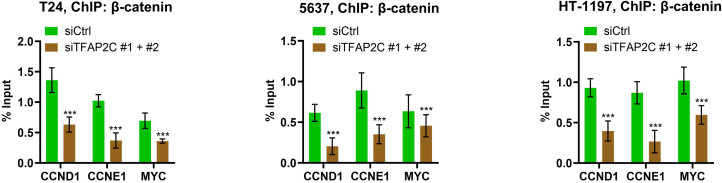


**figs5:**