# Supplementary material for: Menin facilitates the cell proliferation of bladder cancer via modulating the TFAP2C/β-catenin axis
Source: Genes Dis. 2025 Feb 20;12(6):101565. doi: 10.1016/j.gendis.2025.101565 (PMC12361991; doi:10.1016/j.gendis.2025.101565)
Supplement: Multimedia component 1 [file mmc1.docx]

***Supplementary Figure Legends***

**Fig. S1. Significance analysis results for data mining.**

**Fig. S2. Menin regulates TFAP2C transcription mediated by MLL complex in BLCA cells.**

(A, D) ChIP-qPCR assay to assess the levels of menin, H3K4me3, MLL1 or ASH2L recruitment to the TFAP2C promoter in siCtrl- or siMEN1#1 + #2-transfected BLCA cells. ns, non-significant, ** P < 0.05, ** P < 0.01, *** P < 0.001 vs control*.

**Fig. S3 *CTNNB1* dose not regulate TFAP2C expression in BLCa cells.**

RT-qPCR (A) or western blotting (B) showing the expression of TFAP2C in CTNNB1-KD T24, 5637 or HT-1197 cells. ns, non-significant, ** P < 0.05, ** P < 0.01, *** P < 0.001 vs control*.

**Fig. S4.TFAP2C-KD significantly reduced the regulation of β-catenin on the transcription of its downstream target genes.**

ChIP-qPCR assay to assess the levels of β-catenin recruitment to the CCND1, CCNE1 and MYC promoter in siCtrl or siTFAP2C-treated 5637 and HT-1197 cells. ns, non-significant, ** P < 0.05, ** P < 0.01, *** P < 0.001 vs control*.

**Fig. S5. To evaluate the biosafety of BAY-155 or DMSO treatment in a nude mouse tumor-bearing model.**

Representative images for hematoxylin-eosin (HE) staining to analyze the liver, kindey, lung and heart tissues upon the treatment of DMSO or BAY-155 in xenografted-nude mice model.
