# Supplementary material for: Menin facilitates the cell proliferation of bladder cancer via modulating the TFAP2C/β-catenin axis
Source: Genes Dis. 2025 Feb 20;12(6):101565. doi: 10.1016/j.gendis.2025.101565 (PMC12361991; doi:10.1016/j.gendis.2025.101565)
Supplement: Multimedia component 2 [file mmc2.docx]

***Supplementary Table***

**Supplementary Table S1.** Primers used for this study

| **Purpose** | **Primers’name** | **Sequence** | **Reference** |
| --- | --- | --- | --- |
| **RT-qPCR** | MEN1 | F: 5′-GACCTGTCCCTCTATCCTCG-3′ | [1] |
|  |  | R: 5′-TGACCTCAGCTGTCTGCTCC-3′ |  |
|  | TFAP2C | F: 5′-TCAGTCCCTGGAAGATTGTCG-3′ | [2] |
|  |  | R: 5′-CCAGTAACGAGGCATTTAAGCA-3′ |  |
|  | CTNNB1 | F: 5′-TCCTGAGGAAGAGGATGTGGAT-3′ | [3] |
|  |  | R: 5′-CCTCTGAGCTCGAGTCATTGC-3′ |  |
|  | CDK2 | F: 5′-GCTAGCAGACTTTGGACTAGCCAG-3′ | [4] |
|  |  | R: 5′-AGCTCGGTACCA CAGGGTCA-3′ |  |
|  | CDK4 | F: 5′-CTGGTGTTTGAGCATGTAGACC-3′ | [5] |
|  |  | R: 5′- GATCCTTGATCGTTT CGGCTG-3′ |  |
|  | CCND1 | F: 5′-ACGAAGGTCTGCGCGTGTT-3′ | [6] |
|  |  | R: 5′-ACAAACTCTGCTACTTCTGGG-3′ |  |
|  | CCNE1 | F: 5′-ATCAGCACTTTCTTGAGCAACA-3′ | [6] |
|  |  | R: 5′-TTGTGCCAAGTAAAAGGTCTCC-3′ |  |
|  | MYC | F: 5′-TCTGGATCACCTTCTGCTGG-3′ | [7] |
|  |  | R: 5′-AGGATAGTCCTTCCGAGTGG-3′ |  |
|  | ACTB | F:5′-GCAAAGACCTGTACGCCAACA-3′ | [8] |
|  |  | R: 5′-TGCATCCTGTCGGCAATG-3′ |  |
| **ChIP-qPCR** | MBS-1 | F: 5′-TTGGGACAGGGGAGGATACCAG-3′ | |
|  |  | R: 5′-CAAGGAGCTCTTATAAGTCG-3′ | |
|  | MBS-2 | F: 5′-CAGCAGGGAGTGTGCGGCACAG -3′ | |
|  |  | R: 5′-GGTCGGGTTCAGCGCCCGCGGC-3′ | |
|  | MBS-3 | F: 5′-AGTCCTGCAGCTGCTCTCCC-3′ | |
|  |  | R: 5′-GGGAAGGTGGAAATGGTATGGT-3′ | |
|  | MBS-4 | F: 5′-CAGACTGCTGGGCGGCGCGGGGACT -3′ | |
|  |  | R: 5′-CGCGCAGAAGCCGCTGTATCCTG-3′ | |
|  | Bs-1  (TFAP2C promoter) | F: 5′-CAGGCGCCCGAAAGAGGCGCC-3′ | |
|  |  | R: 5′-CCGCAGGAAGCAGCGCTCCTGG -3′ | |
|  | Bs-2  (TFAP2C promoter) | F: 5′-GCAAAGCCCTGCAGAATATGCT-3′ | |
|  |  | R: 5′-CCTTAGACAGCAGCCAAATCCC-3′ | |
|  | Bs-3  (TFAP2C promoter) | F: 5′-GGGAAAGTTTCCCTTGGAGGACG-3′ | |
|  |  | R: 5′-GACTCTCCCATCCACGGAATGGCGGG-3′ | |
|  | Bs-4  (TFAP2C promoter) | F: 5′-GCTGCATAATAACTGGGCCAAAG-3′ | |
|  |  | R: 5′-GGTAGACACCCAGGTCCCACATG-3′ | |
|  | Bs-5  (TFAP2C promoter) | F: 5′-CCTCAGCCAGCCGCGGGAGATG-3′ | |
|  |  | R: 5′-CTCCGTGGGCACAGCGCGGTC-3′ | |
|  | Bs-6  (TFAP2C promoter) | F: 5′-GCAGGTCTTCGGCTGTCAACG-3′ | |
|  |  | R: 5′-GAACAGTGTGTGGTGGCGATAGCC-3′ | |
|  | Bs-1  (CTNNB1 promoter) | F: 5′-GAGACTGGGCTGCGACCCAGG-3′ | |
|  |  | R: 5′-CCGGCCTTCGAGTACCGCTC-3′ | |
|  | Bs-2  (CTNNB1 promoter) | F: 5′-CACCTTCCGCAGGCCGCGG-3′ | |
|  |  | R: 5′-GCTGCGCGGGCCTGAGGGCAG-3′ | |
|  | Bs-3  (CTNNB1 promoter) | F: 5′-CTGCCCTCAGGCCGCGCAGC-3′ | |
|  |  | R: 5′-CAGTCTGCTGCCGTCTGAGCG-3′ | |
|  | Bs-4  (CTNNB1 promoter) | F: 5′-CGGCAGGATACAGCGGCTTCTG-3′ | |
|  |  | R: 5′-TGCTGCCACAGACCGAGAGG-3′ | |
|  | CDK2 promoter | F: 5′-GGATTCCTTTGCAACGAGATTC-3′ | |
|  |  | R: 5′-CTGAACACTCTTCCCAAGTACCTGC-3′ | |
|  | CDK4 promoter | F: 5′-CAATGACACAAATGAGAGTATG-3′ | |
|  |  | R: 5′-CTCACTACCGGTCTGAAGGCTG-3′ | |
|  | CCND1 promoter | F: 5′-GCTCCCATTCTCTGCCGGGC-3′ | |
|  |  | R: 5′-CTCTGGAGGCTCCAGGACTTGC-3′ | |
|  | CCNE1 promoter | F: 5′-CGCATATGGAAGGGGCGCATG-3′ | |
|  |  | R: 5′-CTGAGTCCTGGGCGGGGACATC-3′ | |
|  | MYC promoter | F: 5′-GATGATTTATACTCACAGGAC-3′ | |
|  |  | R: 5′-GAATTAACTACGCGCGCCTACCA-3′ | |

**Reference:**

1. Jiang XH, Lu JL, Cui B, Zhao YJ, Wang WQ, Liu JM, *et al.* MEN1 mutation analysis in Chinese patients with multiple endocrine neoplasia type 1. Endocr Relat Cancer 2007;14:1073–9. doi: 10.1677/ERC-07-0015.
2. Wang X, Sun D, Tai J, Chen S, Yu M, Ren D, Wang L. TFAP2C promotes stemness and chemotherapeutic resistance in colorectal cancer via inactivating hippo signaling pathway. J Exp Clin Cancer Res. 2018 Feb 13;37(1):27. doi: 10.1186/s13046-018-0683-9.

3.[Shucai Yang](https://pubmed.ncbi.nlm.nih.gov/?term=Yang S[Author]), [Yi Liu](https://pubmed.ncbi.nlm.nih.gov/?term=Liu Y[Author]), [Ming-Yue Li](https://pubmed.ncbi.nlm.nih.gov/?term=Li MY[Author]), [Calvin S. H. Ng](https://pubmed.ncbi.nlm.nih.gov/?term=Ng CS[Author]),[Sheng-li Yang](https://pubmed.ncbi.nlm.nih.gov/?term=Yang Sl[Author]), et al. FOXP3 promotes tumor growth and metastasis by activating Wnt/β-catenin signaling pathway and EMT in non-small cell lung cancer. Mol Cancer. 2017 Jul 17;16(1):124. doi: 10.1186/s12943-017-0700-1.

4. Wu XL, Zheng PS. Undifferentiated embryonic cell transcription factor-1 (UTF1) inhibits the growth of cervical cancer cells by transactivating p27Kip1. Carcinogenesis 2013;34:1660–8. doi: 10.1093/carcin/bgt102.

5. Zhang H, Han R, Ling ZQ, Zhang F, Hou Y, You X, et al. PAQR4 has a tumorigenic effect in human breast cancers in association with reduced CDK4 degradation. Carcinogenesis 2018;39:439–46. doi: 10.1093/carcin/bgx143.

6. Cao L, Zhang P, Li J, Wu M. LAST, a c-Myc-inducible long noncoding RNA, cooperates with CNBP to promote CCND1 mRNA stability in human cells. Ulitsky I, ed. eLife 2017;6:e30433. doi: 10.7554/eLife.30433.

7. Chen Y, Rao X, Huang K, Jiang X, Wang H, Teng L. FH535 Inhibits Proliferation and Motility of Colon Cancer Cells by Targeting Wnt/β-catenin Signaling Pathway. J Cancer. 2017 Sep 12;8(16):3142-3153. doi: 10.7150/jca.19273.

8. Weiqiang Jing, Ganyu Wang, Zhiwei Cui, et al. FGFR3 Destabilizes PD-L1 via NEDD4 to Control T-cell Mediated Bladder Cancer Immune Surveillance. Cancer Research. 2022 Jan 1;82(1):114-129. doi: 10.1158/0008-5472.CAN-21-2362.

**Supplementary Table S2.** Antibodies used for western blotting analysis used in this study.

| **Name** | **Supplier** | **Catalog.** | **Dilution** |
| --- | --- | --- | --- |
| anti-menin | Bethyl | A300-105A | 1:2000 |
| anti-TFAP2C | Abcam | ab218107 | 1:2000 |
| anti-MLL1 | Abcam | ab234435 | 1:2000 |
| anti-ASH2L | Abcam | ab314128 | 1:2000 |
| anti-β-catenin | Abcam | ab224803 | 1:2500 |
| anti-CDK2 | Abcam | ab32147 | 1:2500 |
| anti-CDK4 | Abcam | ab108357 | 1:2500 |
| anti-CyclinD1 | Abcam | ab16663 | 1:2500 |
| anti-CyclinE1 | Abcam | ab33911 | 1:2500 |
| anti-Histone H3 | Abcam | ab1791 | 1:2500 |
| anti-H3K4me3 | Abcam | ab8580 | 1:2500 |
| anti-β-actin | Cell Signaling Tech | #4970 | 1:3000 |
